# Prediction of digital transformation of manufacturing industry based on interpretable machine learning

**DOI:** 10.1371/journal.pone.0299147

**Published:** 2024-03-29

**Authors:** Chen Zhu, Xue Liu, Dong Chen

**Affiliations:** 1 Business School, Yangzhou University, Yangzhou, China; 2 School of Economics, Dongbei University of Finance and Economics, Dalian, China; 3 Postdoctoral Workstation of China Dalian International Economic & Technical Cooperation Group Co., Ltd, Dalian, China; 4 School of Accounting, Dongbei University of Finance and Economics, Dalian, China; 5 China Internal Control Research Center, Dalian, China; Chinese Academy of Sciences, CHINA

## Abstract

The enhancement of digital transformation is of paramount importance for business development. This study employs machine learning to establish a predictive model for digital transformation, investigates crucial factors that influence digital transformation, and proposes corresponding improvement strategies. Initially, four commonly used machine learning algorithms are compared, revealing that the Extreme tree classification (ETC) algorithm exhibits the most accurate prediction. Subsequently, through correlation analysis and recursive elimination, key features that impact digital transformation are selected resulting in the corresponding feature subset. Shapley Additive Explanation (SHAP) values are then employed to perform an interpretable analysis on the predictive model, elucidating the effects of each key feature on digital transformation and obtaining critical feature values. Lastly, informed by practical considerations, we propose a quantitative adjustment strategy to enhance the degree of digital transformation in enterprises, which provides guidance for digital development.

## 1. Introduction

In the context of economic globalization, the economic structure has undergone a shift from being primarily reliant on agriculture and industry to becoming increasingly driven by digitalization. In comparison to traditional economies, the digital economy leverages automation and digital tools to enhance efficiency and mitigate costs, enabling businesses to rapidly adapt to market fluctuations and emerging trends. Consequently, the effective development of the digital economy has emerged as a pivotal concern for enterprises [1]. In the current era marked by the growing convergence of the digital economy and the physical economy, the digital transformation of enterprises has emerged as a pivotal catalyst for augmenting their competitive edge within the digital sphere [2], and it has garnered increasing attention as a research hotspot within the academic community [3]. Presently, scholarly research concerning the digital transformation of enterprises primarily concentrates on comprehensively examining the effects of digitalization on pivotal aspects such as innovation capabilities [1, 4], corporate value [5–7], capital markets [8] and sustainable development [9, 10]. Ciampi et al. [11] analyzed the impact of digital transformation on business innovation models by using data from 253 UK companies. Their research findings highlighted that digital transformation can enhance the innovation capabilities of firms by influencing their operational strategies and objectives. Similarly, Ferreira et al. [12] demonstrated that top management can leverage new digital decision-making processes to improve competitiveness through digital transformation. Meanwhile, Zhong et al. [9] investigated the effects of digital transformation on Environmental, Social, and Governance (ESG) factors, revealing a significant enhancement in ESG performance through digital transformation. However, these studies only demonstrate the benefits of digital transformation on firm development. As such, it is crucial to explore ways to enhance digital transformation capabilities at the firm level, thereby promoting digital development within enterprises.

Machine learning, an emerging technology in the field of computer science, empowers computers to acquire knowledge from data, accomplish model training, and perform classification and prediction on novel datasets based on previous acquired knowledge. Within the domain of economics, machine learning has witnessed extensive implementation[13–15]. CraJa et al. [16] used deep learning to analyze the text in the company’s year-end report, and more accurately judged whether the company had financial fraud through the context. Mark et al. [17] utilized machine learning algorithms to predict the presence of financial irregularities in Vietnamese listed companies. They recommend that regulatory bodies should intensify their focus on the financial statements of companies ranked lower in order to detect any potential anomalies.

Currently, China’s manufacturing industry confronts the predicament of high input, high energy consumption, low efficiency, and low output. To address these challenges, digital transformation emerges as a viable solution, enabling companies to effectively mitigate such issues. Hence, this article adopts Chinese listed manufacturing companies as a case study to investigate the impact of various indicators on digital transformation through machine learning. The study further puts forth adjustment strategies aimed at bolstering the capability of digital transformation and accelerating the digitalization process.

## 2. Data and methods

### 2.1 Data set construction

This study aimed to investigate the Digital Transformation Capability (DCG) of listed companies in the A-share market of China’s Shanghai and Shenzhen Stock Exchanges from the year 2014 to 2021. A comprehensive dataset was constructed by selecting companies from the CSMAR [18] and CRNDS [19] databases, resulting in 22,776 initial observational samples. CSMAR and CRNDS are an authoritative database for the Chinese securities market, providing multidimensional research and regulatory data. Specific data sets can be found in S1 Dataset. These samples represented a total of 5,072 listed companies, forming the basis of the study. In order to ensure data quality, missing values were excluded, and a tailing treatment was employed to manage outliers for continuous variables, using the 1st and 99th percentiles. Moreover, focusing specifically on the manufacturing industry, data from sectors C1, C2, C3, and C4 were extracted based on industry classification, resulting in a final dataset comprising 12,057 samples. The meanings of C1, C2, C3 and C4 will be provided in S1 File.

Digital Capability Generation (DCG), refers to the transformative process in which companies harness the potential of digital technology and the internet to revolutionize their business models, processes, organizational structures, and corporate cultures. The ultimate goal is to foster business innovation and optimize operational efficiency. Nevertheless, there is currently no standardized framework for quantitatively evaluating DCG initiatives. In this research endeavor, the application of Python web scraping techniques facilitated the extraction of pertinent keywords, including “artificial intelligence technology,” “blockchain technology,” “cloud computing technology,” “big data technology,” and “digital technology application,” from the annual reports of companies within the designated dataset [12]. The resulting keyword occurrences served as vital indicators to gauge the extent of DCG implementation across these enterprises. Moreover, to ensure a more uniform distribution of DCG data, a logarithmic transformation was applied to the word frequency measurements, as outlined in Formula (1).

DCG=ln(1+N) (1)

Where N represents the number of occurrences of the keyword

**Fig 1:**
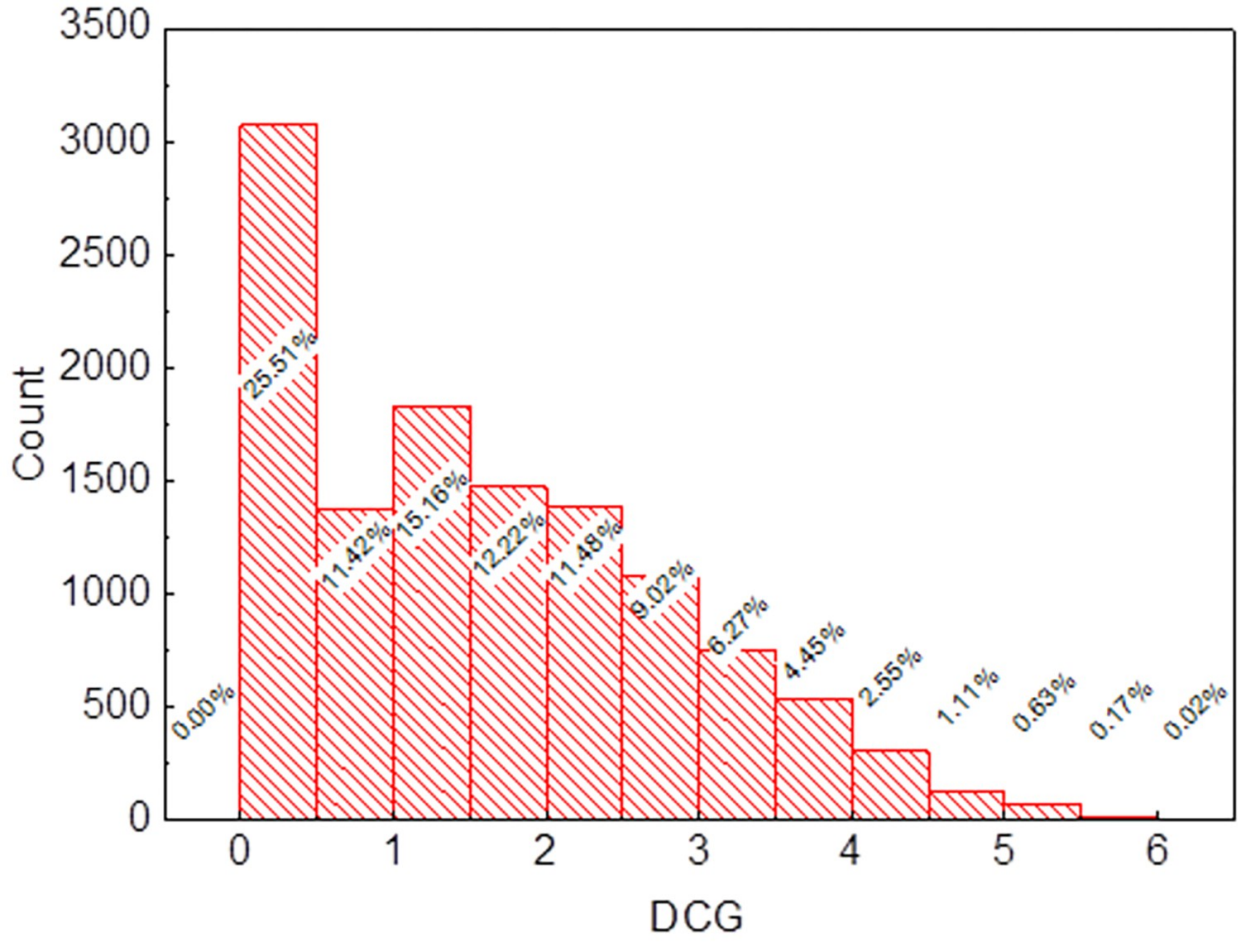
The distribution of DCG in the dataset.

Fig 1 depicts the distribution of DCG within the dataset, revealing that more than 99% of the samples exhibit a DCG value lower than 5. This finding underscores the presence of a notable imbalance issue among the samples. To enhance the efficacy of subsequent predictions and bolster the model’s generalization capacity, a threshold of 1.5 was utilized for DCG. Any DCG value below 1.5 indicated a lower level of digital transformation for the enterprise and correspondingly received a label of 0. Conversely, DCG values exceeding 1.5 denoted a higher degree of digital transformation, resulting in a label of 1. As a result, the final dataset comprised 6,280 samples labeled as 0 and 5,777 samples labeled as 1.

### 2.2 Feature engineering

To establish a predictive model for DCG, a range of enterprise information features were selected and presented in Table 1. Specifically, the features were categorized into financial and non-financial indicators. The former comprised a company’s development ability, debt-paying ability, profitability, cash acquisition ability, and operational ability, whereas the latter consisted of corporate governance, equity structure, financing capability, company size, years since its establishment, and company value.

**Table 1:** Feature name and description.

Features	Type	Name	Symbol	Description
Financial indicators	Development ability	Proportion of R&D personnel	RDpr	Total number of R&D personnel/employees
		The proportion of R&D expenditure to operating income.	RDeapoinr	R&D expenditure/operating income
		Capitalized R&D expenditure as a percentage of R&D expenditure	CapRDexpr	Capitalized R&D expenditure as a proportion of R&D expenditure
		Revenue growth rate	Growth	Current year operating income/Previous year operating income -1
	Solvency	Asset-liability ratio	Lev	Total liabilities/total assets
		Loss situation	Loss	If the net profit is less than 0, the value is 1, otherwise the value is 0
	Profitability	Net profit rate on total assets	ROA	Net profit/average balance of total assets
	Cash earning capacity	Cash flow ratio	Cashflow	Net cash flows from operating activities/total assets
	Management ability	Turnover of total assets	ATO	Operating income/Average assets
Non-financial indicators	Corporate governance	Number of directors	Board	Take the natural logarithm of the number of board members
		Proportion of independent directors	Indep	Number of independent directors/Total number of board members
		Equity balance degree	Balance1	The second largest shareholder divided by the first largest shareholder
	Ownership structure	The proportion of the largest shareholder	Top1	Number of shares held by the largest shareholder/total number of shares
		Property right nature	SOE	For state-owned holding enterprises, the value is 1; otherwise, the value is 0
		Proportion of shareholding by institutional investors	INST	Total shares held by institutional investors/total share capital
		Management shareholding ratio	Mshare	Management shareholding figure divided by total equity
	Financing capacity	Financing constraint	SA	SA = -0.737size+0.043size^2–0.04FirmAge
	Company size	Company size	Size	Take the natural log of total assets
	Years of establishment	Years of establishment	FirmAge	ln(Year of the year—year of establishment +1)
	Company value	Tobin’s Q value	TobinQ	(Market value of tradable shares + number of non-tradable shares × net assets per share + book value of liabilities)/ total assets

### 2.3 Machine learning model

#### 2.3.1 Algorithm introduction

Machine learning algorithms can be categorized into regression and classification based on the type of the target variable. This article focuses on predicting DCG values of 0 or 1, which classifies it under the classification algorithms. In classification algorithms, the model is trained using the features and labels of the sample data to predict unknown data. To assess the performance of different algorithm types on the dataset, this study selects extreme random trees (ETC) [20] as representative of the Bagging algorithm, gradient boosting machines (GBM) [21] as representative of the Boosting algorithm, support vector machines (SVM) [21] as representative of hyperplanes, Logistic regression (LOG), and Multi-layer perceptron (MLP) [22, 23] as representative of neural networks. The principle of the specific algorithm will be provided in the S1 File.

#### 2.3.2 Model validation and evaluation indicators

In order to mitigate overfitting during the training process, this study utilizes cross-validation and holdout methods to establish the model. The dataset is randomly partitioned into a training set and a testing set in an 8:2 ratio. The model is trained on the training set, while the testing set is used to assess the model’s generalization ability. To minimize the model’s generalization error, a 5-fold cross-validation approach is employed during the training process [24].

In the context of classification models [25], particularly binary classification models, performance is often assessed using a confusion matrix. The confusion matrix displays the model’s classification predictions and includes the following metrics: True Positive (TP), False Positive (FP), True Negative (TN), and False Negative (FN), as outlined in Table 2. These metrics serve as the basis for computing evaluation metrics such as Accuracy, Precision, Recall, and F1 score, which are determined through Formulas (2)–(5).

**Table 2:** Binary confusion matrix.

Real situation	Forecast result	
	Positive instance	Negative instance
Positive instance	True positive instance (TP)	True negative instance (FN)
Negative instance	False positive instance (FP)	False negative instance (TN)


$$Accuracy=\frac{TP+TN}{TP+FN+FP+TN}$$ (2)

$$\mathrm{Pr}ecision=\frac{TP}{TP+FP}$$ (3)

Recall=TPTP+FN (4)

f1=2×Precision×RecallPrecision+Recall (5)

To evaluate the effectiveness of various classification models further, the Receiver Operating Characteristic (ROC) curve is used to compare their generalization capabilities [26, 27]. The ROC curve demonstrates the relationship between True Positive (TP) and False Positive (FP) rates for a specified classifier. The ROC curve plot displays the horizontal axis as the FP rate, while the vertical axis represents the TP rate. Each point on the ROC curve corresponds to the TP rate and FP rate for a particular threshold. A classifier with an ROC curve that is closer to the upper left corner signifies superior performance. A larger area under the ROC curve indicates better model performance.

#### 2.3.3 Hyperparameter optimization

After determining the best machine learning algorithm, optimizing the hyperparameters within the algorithm becomes necessary. Presently, common methods for hyperparameter optimization are grid search, random search, and Bayesian optimization [28, 29]. Grid search is a systematic approach to hyperparameter tuning. It involves defining the range of potential hyperparameter values to be explored, and then exhaustively evaluating all possible combinations of parameters using cross-validation or other evaluation metrics. Ultimately, the optimal hyperparameters are selected based on the highest observed performance. The main advantage of grid search is its ability to find the global optimum, but the downside is the high computational cost, particularly when dealing with a large number of parameters. Random search, on the other hand, is a method that randomly selects hyperparameters within a given range. By specifying the number of iterations or setting a stopping criterion, relatively good hyperparameters can be discovered within a specific time frame or number of iterations. Compared to grid search, random search is advantageous due to its simplicity and computational efficiency. However, since the parameters are selected randomly, there is no guarantee of finding the global optimum.

Bayesian Optimization is a sequential model optimization method based on Bayesian inference [30–32]. It is used to optimize the input parameters of a black-box function. Compared to grid search and random search, Bayesian Optimization can efficiently identify the optimal parameters. The fundamental concept of Bayesian Optimization is to establish a prior model, which estimates the parameter performance using observed parameters and their corresponding function values. Generally, the prior model assumes that the function values follow a Gaussian process and updates the parameter model through Bayesian inference. In each iteration, the next parameter with the highest likelihood of achieving better performance is selected for evaluation based on the predicted results of the parameter model. By repeating this process, Bayesian Optimization gradually converges to the global optimum. Considering the large dataset and complex range of hyperparameters in this paper, Bayesian Optimization is chosen as the approach for hyperparameter optimization. Accuracy is used as the evaluation metric, and the best hyperparameter combination is obtained through iterative optimization. Table 3 presents the hyperparameters obtained for the four machine learning models after applying Bayesian Optimization.

**Table 3:** Hyperparameters of machine learning model obtained by Bayesian optimization.

Model	Hyperparameter	Search range	Optimal hyperparameter
ETC	n_estimators	[10,2000]	2000
	max_depth	[2,100]	58
	min_samples_split	[2,50]	2
	min_samples_leaf	[1,50]	1
	max_features	[0.1,1]	0.25
GBM	n_estimators	[50,2000]	1247
	learning_rate	[0.001,0.1]	0.056
	max_depth	[2,20]	16
	subsample	[0.5,1.0]	0.81
	min_samples_split	[2,50]	2
	min_samples_leaf	[1,20]	1
SVM	C	[10,5000]	1576
	gamma	[0.1,20]	0.1
LOG	C	[0.01,100]	1
	penalty	‘l1’,‘l2’	‘l1’
	sovler	’newton-cg’, ’lbfgs’, ’liblinear’, ’sag’, ’saga’	‘liblinear’
MLP	hidden_layer_sizes	[10,1000]	500
	learning_rate_init	[0.0001,0.01]	0.0042
	max_iter	[10,1000]	908
	alpha	[0.0001,0.01]	0.0001

## 3. Results and discussion

### 3.1 Model establishment

**Fig 2:**
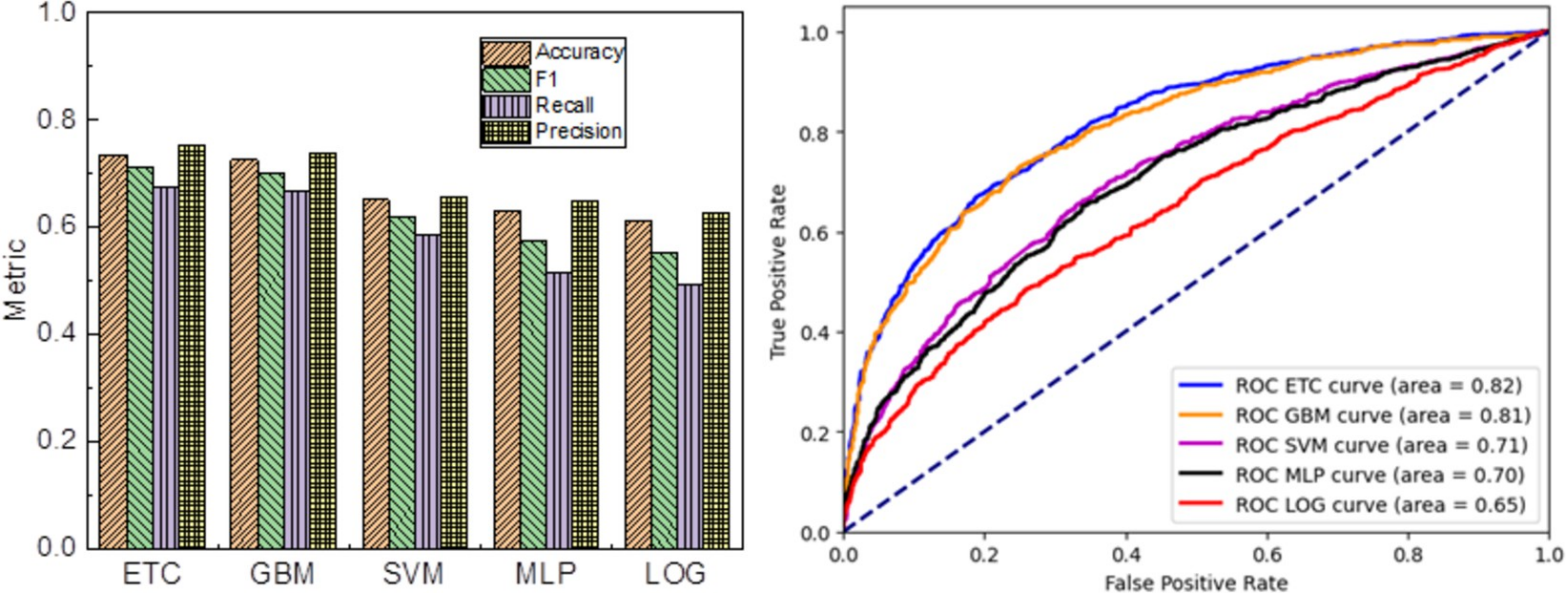
Comparison of four classification models on test sets. (a) Evaluation indicators, (b) ROC.

Fig 2 illustrates the performance of the four machine learning models on the test dataset. In Fig 2(A), an evaluation metrics comparison is presented for these models. Notably, the ETC and GBM models outperform the SVM and MLP models. The ETC model achieves an accuracy of 0.74, an F1 score of 0.72, a recall of 0.67, and a precision of 0.75. The ROC curves for the four machine learning models are displayed in Fig 2(B). It is evident that the ROC curve of the ETC model almost entirely encompasses the curves of the other three models, depicting its superior performance. Furthermore, the ETC model demonstrates the largest AUC area of 0.82 among all the models, further affirming its optimal predictive accuracy. Consequently, the subsequent analysis will be carried out utilizing the ETC model.

### 3.2 Feature screening

**Fig 3:**
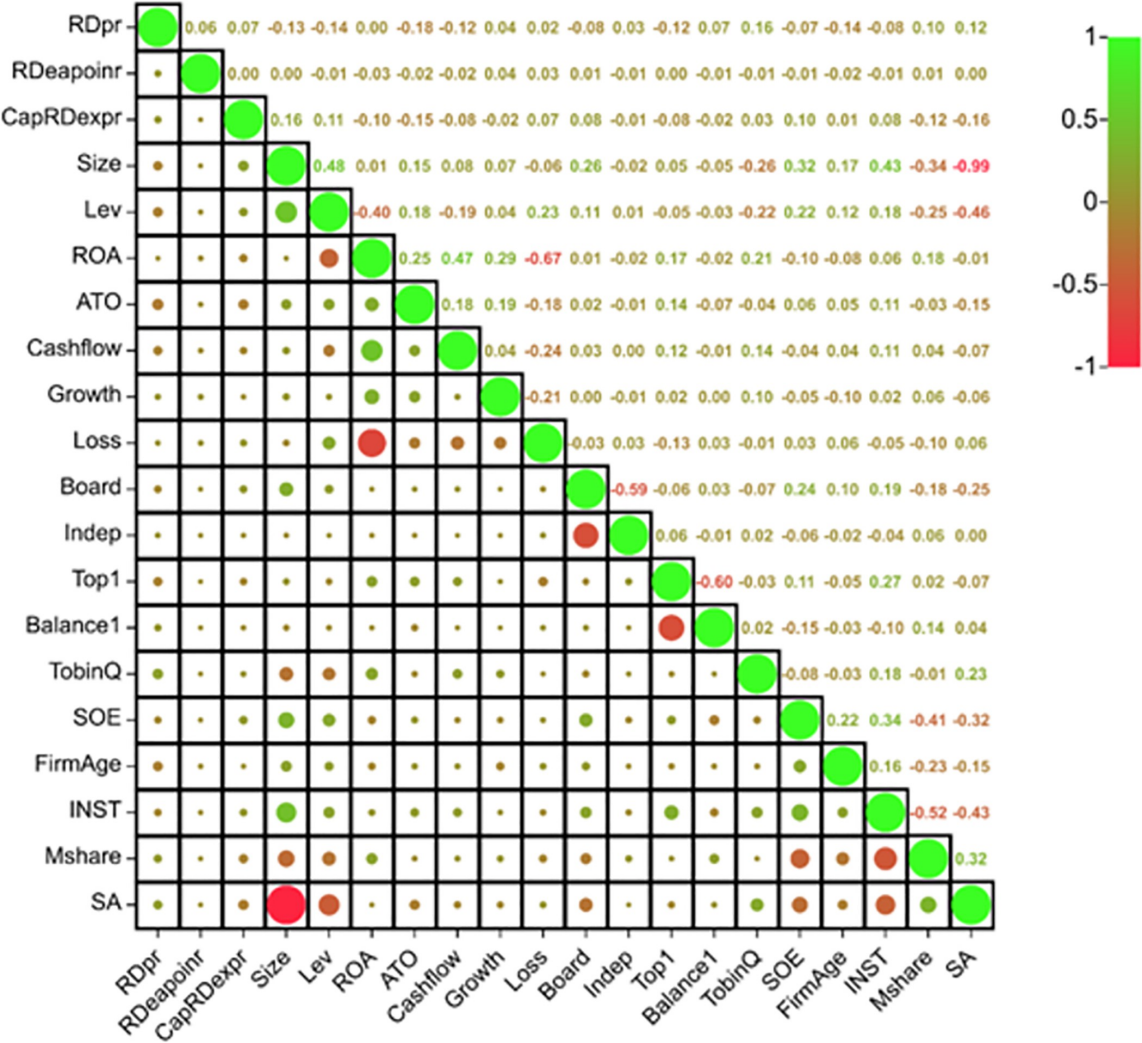
Correlation heat map between features.

Since the feature redundancy was not taken into account during the feature selection process, it is necessary to refine the dataset after selecting the optimal algorithm. Fig 3 presents the heatmap of feature correlations. When two features have a correlation greater than 0.95 [33, 34], it suggests a high degree of correlation, and one of the features may be removed. In Fig 6, the correlation coefficient between Size and SA is 0.99, indicating that Size can be removed, leaving 19 remaining features. Recursive Feature Elimination (RFE) and Exhaustive Feature Selection (EFS) are further implemented for feature selection. RFE is an embedded feature selection approach, which trains a model on the original dataset and iteratively eliminates low-weight features to yield a feature subset. Specifically, the method trains a model on the original dataset, ranks the features based on their weights. Then, it iteratively removes the feature with the smallest weight and trains a model on the remaining features.

**Fig 4:**
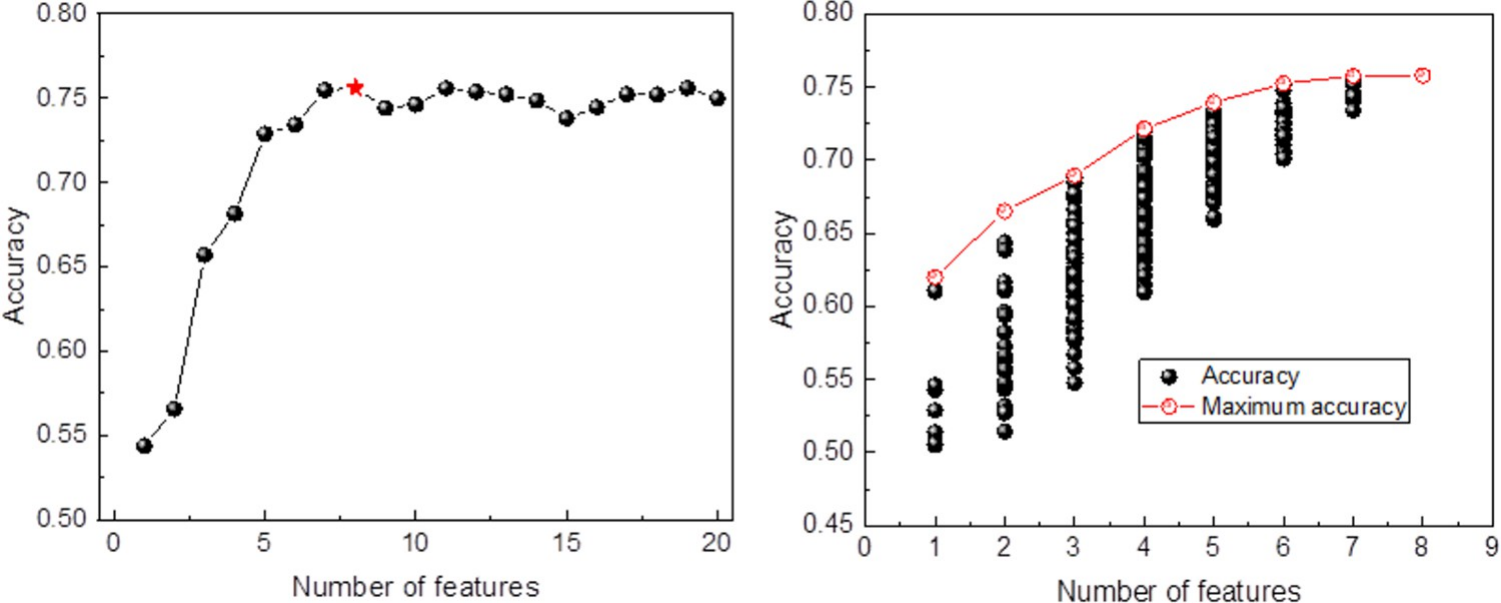
Feature selection process (a) recursive elimination (b) exhaustion method.

Based on the models’ performance in each iteration, the optimal feature subset and its corresponding model were chosen. The process of feature selection using the recursive elimination method is depicted in Fig 4(A). As the number of features in the subset increased, the model’s accuracy continually improved, reaching 0.756 when there were 8 features. Beyond 8 features, no further improvement was observed. Thus, 8 was identified as the best feature subset, including RDpr, RDeapoin, Lev, ATO, Top1, Balance1, Mshare, and SA. The exhaustive method exhaustively explores all feature combinations within the subset and evaluates the model’s performance for each combination. Fig 4(B) illustrates the feature selection process utilizing the EFS. Among all feature combinations, the combination of 8 features achieved the highest accuracy, confirming RDpr, RDeapoin, Lev, ATO, Top1, Balance1, Mshare, and SA as critical features.

### 3.3 Interpretability analysis

Although machine learning has high prediction accuracy, its inherent prediction process is still invisible and belongs to the field of black box model. To enhance the interpretability of models, this study introduces Shapley Additive Explanation (SHAP) values for analysis [30]. SHAP values serve as a method for elucidating the significance of features within predictive models. In the context of machine learning, SHAP values provide insight into the relative contributions that each feature makes to the model’s predictions. This is achieved through the permutation and combination of input features, assigning them weights according to their influence on the predicted outcome, thereby producing SHAP values for individual features. Analyzing these SHAP values facilitates the extraction of valuable information about feature importance rankings and feature interactions within the model, thereby aiding in our understanding and explication of the decision-making processes employed by the model. The specific explanation of SHAP value is in S1 File.

**Fig 5:**
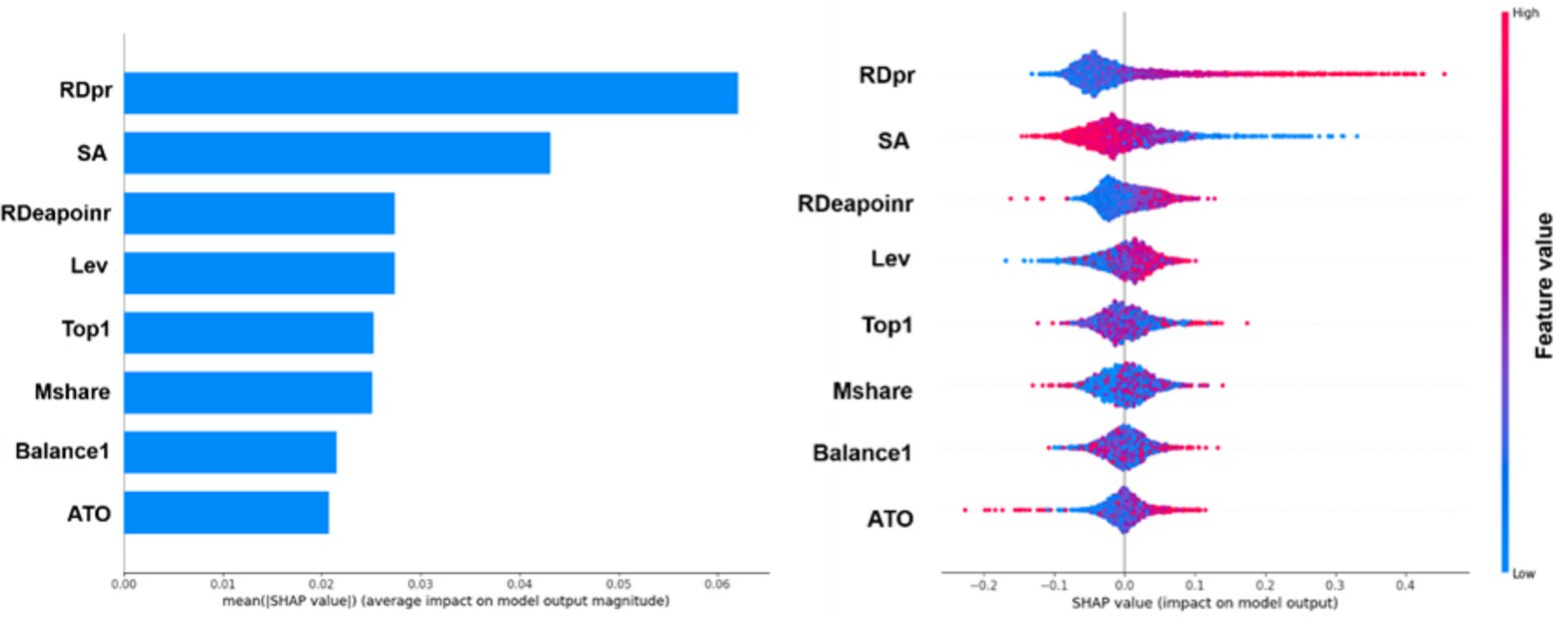
Characteristic summary of SHAP values. (a) Average SHAP values (b) SHAP values for each sample.

Fig 5 depicts the feature summary plot of SHAP values in a manner consistent. Fig 5(A) showcases the distribution of average SHAP values for each feature. The y-axis signifies the different features, while the length of the bars represents their respective significance. The ranking of feature importance is as follows: RDpr, SA, RDeapoinr, Lev, Top1, Mshare, Balance1, and ATO. Fig 5(B) portrays the distribution of SHAP values for samples in the test set. On the x-axis, positive and negative values denote a positive or negative impact on the target variable, respectively. The y-axis corresponds to different features, with each point representing a distinct sample. The color of each point spans from red to blue, with red indicating a higher feature value and blue indicating a lower feature value. For instance, regarding the RDpr feature, the red points largely concentrate in the positive half of the x-axis, while the blue points are spread across the negative half. This indicates that an increase in RDpr values promotes the transition of the class towards 1, thereby enhancing the level of digital transformation.

**Fig 6:**
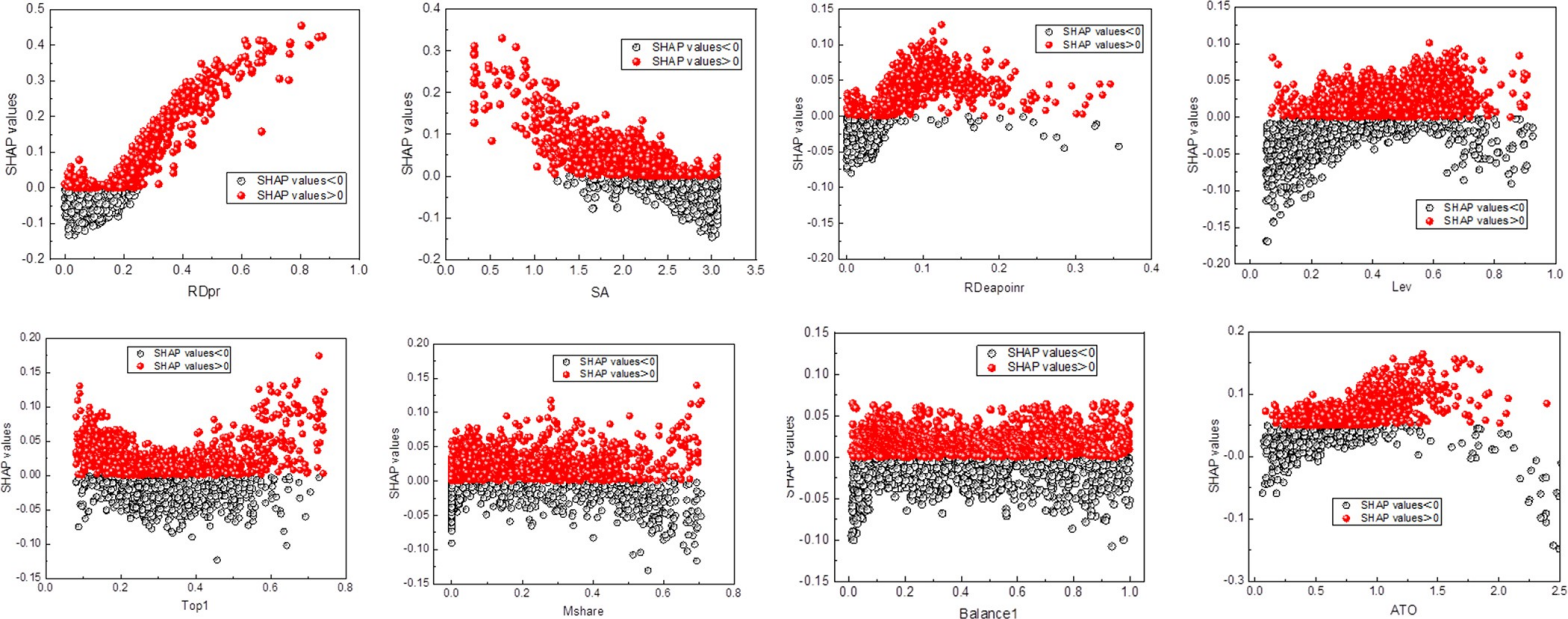
The SHAP dependence plot for (a) RDpr (b)SA (c) RDeapoinr (d) Lev (e) Top1 (f) (g) MshareBalance1 (h) ATO.

The SHAP values of each feature were plotted according to their positive or negative contributions, as depicted in Fig 6. Analyzing Fig 6(A) and 6(C), it is evident that when RDpr > 0.25 and RDeapoinr > 0.05, the majority of SHAP values for the samples are positive. This indicates a positive impact on the target variable and facilitates digital transformation. RDpr and RDeapoinr represent the proportions of research and development personnel and research and development expenses to operating revenue, respectively. These factors significantly influence a company’s digital transformation [35]. A higher ratio of research and development personnel and investment supports the organization in undertaking more technological innovation and product development. This, in turn, helps companies maintain a technological advantage and introduce more competitive digital products and services. Moreover, research and development personnel and expenses play a pivotal role in safety and risk management. A greater proportion of research and development personnel and spending enables companies to develop and implement comprehensive security strategies, guaranteeing data and system safety throughout the digital transformation process [36, 37].

Fig 6(B) illustrates that when SA > 2.5, the SHAP values for the samples are predominantly negative, which hampers an enterprise’s digital transformation progress.

SA represents the financial constraints of the enterprise. A larger SA indicates potential funding shortages for the company. Digital transformation, on the other hand, necessitates substantial investments, particularly in technology research and development, data analysis, and automation, which demand significant resources and funding [38]. When confronted with financial constraints, the company’s digital transformation initiatives may face limitations, impeding the pace and effectiveness of the transformation [39]. Fig 6(D) depicts the distribution of SHAP values corresponding to Lev. In the range of 0.3 < Lev < 0.7, the majority of SHAP values for the samples are positive, indicating beneficial conditions for the enterprise’s digital transformation. Conversely, negative values are detrimental to the digital transformation of the enterprise. Lev, representing the leverage ratio of an enterprise, has a dual impact on digital transformation. Firstly, digital transformation entails significant financial investments. In the case of a low debt ratio, the company may encounter a shortage of initial funding, impeding the smooth execution of digital transformation plans [40]. Conversely, companies with a high debt ratio must address management contracts and debt repayment concerns. Consequently, the scale of investment in digital transformation may be constrained, leading to adverse effects on the speed and efficacy of the transformation [41]. Further, companies with a high debt ratio tend to prioritize cost control, resulting in relatively limited investment in digital transformation and innovation. This, in turn, influences their digital capabilities and competitiveness. Conversely, companies with a moderate debt ratio emphasize management efficiency by effectively distributing resources and assets, thereby achieving a more efficient digital transformation.

Fig 6(E)–6(G) depict the distribution of SHAP values corresponding to Top1, Mshare, and Balance1, respectively. When Top1 is less than 0.2 or greater than 0.6, SHAP values tend to be positive, which is advantageous for digital transformation. Top1 represents the shareholding proportion of the foremost stakeholder in the company. A higher shareholding proportion for the primary stakeholder typically signifies greater capacity for resource investment, encompassing both funding and technological resources. Consequently, the primary stakeholder can allocate resources more flexibly to support the indispensable technological and equipment investments required for digital transformation. Moreover, a higher proportion of shares held by the primary stakeholder generally implies a stronger influence on corporate decision-making. In the context of digital transformation, a series of strategic decisions and transformative measures must be undertaken [42]. A higher proportion of shares held by the largest shareholder ensures a greater influence and control over decision-making relating to digital transformation, thereby facilitating a smooth transition. Conversely, a lower proportion of shares held by the largest shareholder may prompt the company to prioritize effective management mechanisms and processes. To attract investment and support from other shareholders, the organization may strengthen internal management and enhance efficiency. This approach is advantageous for driving digital transformation as it typically requires efficient organization and processes. Simultaneously, a smaller proportion of shares held by the largest shareholder may suggest a more open attitude towards external investments. Attracting external investment can bring fresh funding, technology, and market resources to the company, thus expediting the progress of digital transformation. The concept of Mshare, which represents the ownership percentage of shares by management, exerts a multifaceted influence on the digital transformation of enterprises. On the one hand, a significant hold share by management empowers them to enhance their sway and control over corporate decision-making. This may foster a proactive approach to driving decisions and executing digital transformation initiatives, thereby expediting decision-making and improving execution efficiency. On the other hand, an elevated management hold share may lead to an immoderate concentration of power within the organization, consequently creating a deficiency in effective oversight and counterbalancing mechanisms. Such circumstances potentially pave the way for excessive managerial centralization, amplifying the shortage of appropriate feedback and constraints, posing a risk to the quality and efficacy of digital transformation decision-making [43]. The term Balance1 refers to the degree of equity balance in a company. An excessively high degree of equity balance suggests that power is dispersed among multiple shareholders, each with their individual opinions and vested interests. This situation can potentially lead to slow and intricate decision-making processes that require more negotiations and compromises, thereby impeding the progress of digital transformation projects. Conversely, a deficiency of equity balance can result in the interests of other shareholders or stakeholders being neglected [44, 45], which may prompt decision-making and the implementation of digital transformation initiatives without fully considering the interests and objectives of the whole company. Consequently, this may weaken the overall competitiveness and sustainability of the company in the long run.

Fig 6(H) demonstrates the SHAP value distribution of ATO, indicating that ATO > 0.7 is advantageous for a company’s digital transformation. ATO represents the total asset turnover ratio, an important metric for assessing a company’s efficiency in asset operation. It reflects the relationship between sales generated through operational activities and the total assets of the company within a specific time period. A higher total asset turnover ratio signifies greater efficiency in asset operation, enabling the company to utilize assets more effectively to drive sales [46]. Considering that digital transformation typically requires substantial investment, a high turnover ratio can free up and redirect funds, providing additional resources to support the technological and equipment investments needed for digital transformation [47]. Moreover, a high total asset turnover ratio also necessitates the company’s ability to quickly adapt to market demand changes and flexibly adjust production and sales strategies. Digital transformation can provide more comprehensive data and analysis resources, enabling the company to make more precise market forecasts and informed decisions. By enhancing a company’s innovative capabilities and competitiveness through digital transformation, the total asset turnover ratio can be further improved.

### 3.4 Improve quantitative adjustment strategies for digital transformation

Based on our predictive model and interpretability analysis, we propose a quantitative strategy to enhance companies’ digital transformation [48]. Among the eight key features, namely RDeapoinr, Lev, and ATO, adjusting the latter three is relatively easier compared to the remaining features, which are relatively fixed. Therefore, in practical implementation, while maintaining the other features unchanged, adjustments can be made to RDeapoinr, Lev, and ATO in order to transform companies originally labeled as 0 to label 1.

In the process of customizing the subsequent development direction, companies need to consider not only the predictive results of the model but also additional factors like cost and efficiency. Consequently, we propose an adjustment model, represented by Formulas (6)~(9). More specifically, we constrain the values of RDeapoinr, Lev, and ATO to fall within the critical value range proposed in section 3.3, which is defined as 0.05 < RDeapoinr < 0.4, 0.3 < Lev < 0.7, and 0.7 < ATO < 2.5, while the remaining features remain fixed. These specific values are then substituted into predictive model (6) to derive the corresponding predicted labels. We select the set of predicted labels with a value of 1, and based on the constraints outlined in (7), (8), and (9), we obtain the final adjusted feature values.

P=Model(RDeapoinr,Lev,ATO,F) (6)

$$RDeapoinr^{*}=Min(RDeapoinr)$$ (7)

$$Lev^{*}=\frac{Min(Lev)+Max(Lev)}{2}$$ (8)

$$ATO^{*}=Max(ATO)$$ (9)

Where *P* represents the predicted label, *Model* represents the established predictive model, RDeapoinr, Lev, and ATO correspond to the respective features, F represents the remaining unchanged features, and RDeapoinr*, Lev*, and ATO* represent the adjusted feature values.

This paper presents a case study of two listed companies with stock codes 000004 and 000017 in 2016, aimed at demonstrating how to enhance digital transformation through adjusted strategies, as illustrated in Table 4. The company with stock code 000004 started with RDeapoinr, Lev, and ATO values near their respective critical points. Subsequent feature adjustments led to increases in these metrics, with the corresponding label changing from 0 to 1. In contrast, the company with stock code 000017 started with higher Lev and ATO values, but low RDeapoinr values. By increasing the proportion of RDeapoinr and lowering Lev and ATO levels based on the company’s actual conditions, the label was eventually transformed. (The adjusted values given are not absolute, but only provide reference for improvement).

**Table 4:** Comparison of raw data with adjusted data.

No.		RDeapoinr	Lev	ATO	Label
000004	Original	0.0321	0.2035	0.9244	0
	After adjustment	0.25	0.56	1.4	1
000017	Original	0.0046	0.6805	2.4797	0
	After adjustment	0.11	0.51	1.6	1

## 4. Conclusion

This paper aims to predict the degree of digital transformation in the manufacturing industry using machine learning techniques. It compares four machine learning algorithms and evaluates their performance based on several metrics. The ETC algorithm achieved the highest predictive accuracy in the test set, with an accuracy of 0.74, F1 score of 0.72, recall of 0.67, and precision of 0.75. To identify the most relevant features, the paper conducted correlation analysis and recursive feature elimination. It found that the optimal feature subset includes Proportion of R&D personnel, The proportion of R&D expenditure to operating income, Asset-liability ratio, Turnover of total assets, The proportion of the largest shareholder, Equity balance degree, Management shareholding ratio, and Financing constraint. Additionally, SHAP values were used to analyze interpretability and determine the ranking of feature importance. The most crucial features identified were Proportion of R&D personnel, Financing constraint, The proportion of R&D expenditure to operating income, Asset-liability ratio, The proportion of the largest shareholder, Management shareholding ratio, Equity balance degree, and Turnover of total assets. From a corporate development perspective, it is imperative to increase the proportion of R&D personnel and the ratio of R&D expenses to operating income. R&D personnel typically possess technical expertise and innovation capabilities, enabling companies to adeptly address technological challenges in digital transformation. Firms with a higher ratio of R&D expenses to operating income tend to prioritize technological innovation and invest significantly in research and development, thereby contributing to the sustenance of a competitive advantage in digital transformation. The proportions of R&D personnel and R&D expenses to operating income wield a substantial impact on a company’s digital transformation. Accordingly, companies should judiciously allocate the proportion of R&D personnel and the ratio of R&D expenses to operating income based on their unique circumstances to achieve a successful digital transformation. From the standpoint of the company’s debt-paying ability, there is a need to curtail the asset-liability ratio. The asset-liability ratio serves as a pivotal indicator of a company’s financial health, reflecting the intricate relationship between its assets and liabilities. A diminished asset-liability ratio signifies reduced financial risk, potentially aiding the company in better navigating challenges in digital transformation. In terms of operational capability, the company should strive to enhance its capital turnover rate. The capital turnover rate stands as a vital financial management metric, indicative of the efficiency of a company’s capital utilization and debt-paying ability. A heightened capital turnover rate signifies superior efficiency in capital utilization, thereby assisting the company in effectively addressing challenges in digital transformation. Concerning the company’s equity structure, the impact of the proportion of the largest shareholder’s holdings, management’s shareholding ratio, and equity balance on digital transformation is intricate. Companies should comprehensively consider various factors and formulate the optimal digital transformation strategy based on their individual circumstances. From a financing capability perspective, companies should alleviate financing constraints to expedite the pace and efficacy of digital transformation. This suggests that these conditions are beneficial for digital transformation in enterprises. Based on the findings, the paper proposes a quantitative adjustment strategy for digital transformation, taking into account the actual conditions of the enterprise. This strategy aims to promote the progress of digital development in the manufacturing industry.

## Supporting information

S1 Dataset (CSV)

S1 File (DOCX)

## References

[1] Li R., Rao J., Wan L.,(2022) The digital economy, enterprise digital transformation, and enterprise innovation, Managerial and Decision Economics 43(7) 2875–2886.

[2] Fitzgerald M., Kruschwitz N., Bonnet D., Welch M.,(2014) Embracing digital technology: A new strategic imperative, MIT sloan management review 55(2) 1.

[3] Zhang T., Shi Z.-Z., Shi Y.-R., Chen N.-J.,(2022) Enterprise digital transformation and production efficiency: Mechanism analysis and empirical research, Economic research-Ekonomska istraživanja 35(1) 2781–2792.

[4] Karagiannaki A., Vergados G., Fouskas K.,(2017) The impact of digital transformation in the financial services industry: Insights from an open innovation initiative in fintech in Greece, MCIS Proceedings.

[5] Porter M.E., Heppelmann J.E.,(2015) How smart, connected products are transforming companies, Harvard business review 93(10) 96–114.

[6] Peng Y., Tao C.,(2022) Can digital transformation promote enterprise performance?—From the perspective of public policy and innovation, Journal of Innovation & Knowledge 7(3) 100198.

[7] Du X., Jiang K.,(2022) Promoting enterprise productivity: The role of digital transformation, Borsa Istanbul Review 22(6) 1165–1181.

[8] Hajli M., Sims J.M., Ibragimov V.,(2015) Information technology (IT) productivity paradox in the 21st century, International Journal of Productivity and Performance Management 64(4) 457–478.

[9] Zhong Y., Zhao H., Yin T., Resource Bundling: How Does Enterprise Digital Transformation Affect Enterprise ESG Development?, Sustainability, 2023.

[10] He Q., Ribeiro-Navarrete S., Botella-Carrubi D.,(2023) A matter of motivation: the impact of enterprise digital transformation on green innovation, Review of Managerial Science.

[11] Ciampi F., Demi S., Magrini A., Marzi G., Papa A.,(2021) Exploring the impact of big data analytics capabilities on business model innovation: The mediating role of entrepreneurial orientation, Journal of Business Research 123 1–13.

[12] Ferreira J.J.M., Fernandes C.I., Ferreira F.A.F.,(2019) To be or not to be digital, that is the question: Firm innovation and performance, Journal of Business Research 101 583–590.

[13] Bragoli D., Ferretti C., Ganugi P., Marseguerra G., Zammori F.J.S.E.A.,(2021) Machine-learning models for bankruptcy prediction: do industrial variables matter?, (2) 1–22.

[14] Barboza F., Kimura H., Altman E.,(2017) Machine learning models and bankruptcy prediction, Expert Systems with Applications 83 405–417.

[15] Chen M.-Y.,(2011) Bankruptcy prediction in firms with statistical and intelligent techniques and a comparison of evolutionary computation approaches, Computers & Mathematics with Applications 62(12) 4514–4524.

[16] Craja P., Kim A., Lessmann S.,(2020) Deep learning for detecting financial statement fraud, Decision Support Systems 139 113421.

[17] Lokanan M., Tran V., Vuong N.H.,(2019) Detecting anomalies in financial statements using machine learning algorithm, Asian Journal of Accounting Research 4(2) 181–201.

[18] https://data.csmar.com/, 2022.

[19] https://www.cnrds.com/Home/Login#/BaseDatabase, 2022.

[20] Galar M., Fernandez A., Barrenechea E., Bustince H., Herrera F.,(2011) A review on ensembles for the class imbalance problem: bagging-, boosting-, and hybrid-based approaches, IEEE Transactions on Systems, Man, Cybernetics, Part C 42(4) 463–484.

[21] Xu M., Wang J., Sun Y., Zhu S., Zhang T., Guan S.,(2023) Prediction of glass-forming ability in ternary alloys based on machine learning method, Journal of Non-Crystalline Solids 616 122476.

[22] Hu J., Xu X., Cui Y., Xu M., Gao X., Ji X.,(2023) An ensemble learning based amorphous state predictor for multicomponent alloys, Journal of Non-Crystalline Solids 607 122116.

[23] Cavanaugh M., Buchheit R., Birbilis N.,(2010) Modeling the environmental dependence of pit growth using neural network approaches, Corrosion Science 52(9) 3070–3077.

[24] Pan S., Wang Y., Yu J., Yang M., Zhang Y., Wei H., et al.,(2021) Accelerated discovery of high-performance Cu-Ni-Co-Si alloys through machine learning, Materials and Design 209 109929.

[25] Liu C., Wang X., Cai W., He Y., Su H.,(2023) Machine Learning Aided Prediction of Glass-Forming Ability of Metallic Glass, Processes 11(9) 2806.

[26] Liu G., Sohn S., Kube S.A., Raj A., Mertz A., Nawano A., et al.,(2023) Machine learning versus human learning in predicting glass-forming ability of metallic glasses, Acta Materialia 243.

[27] Ward L., O’Keeffe S.C., Stevick J., Jelbert G.R., Aykol M., Wolverton C.,(2018) A machine learning approach for engineering bulk metallic glass alloys, Acta Materialia 159 102–111.

[28] Liu X., Long Z., Peng L.,(2023) Prediction of Vickers hardness of amorphous alloys based on interpretable machine learning, Journal of Non-Crystalline Solids 602 122095.

[29] Xiong J., Shi S., Zhang T.,(2020) A machine-learning approach to predicting and understanding the properties of amorphous metallic alloys, Materials & Design 187 108378.

[30] Liu C., Wang X., Cai W., Yang J., Su H.,(2023) Optimal Design of the Austenitic Stainless-Steel Composition Based on Machine Learning and Genetic Algorithm, Materials 16(16). doi: 10.3390/ma16165633 37629924 PMC10456822

[31] Hernández-Lobato J.M., Gelbart M.A., Adams R.P., Hoffman M.W., Ghahramani Z.,(2016) A general framework for constrained Bayesian optimization using information-based search, The Journal of Machine Learning Research.

[32] Shahriari B., Swersky K., Wang Z., Adams R.P., De Freitas N.,(2015) Taking the human out of the loop: A review of Bayesian optimization, Proceedings of the IEEE 104(1) 148–175.

[33] Zhang H., Fu H., He X., Wang C., Jiang L., Chen L., et al., (2020) Dramatically enhanced combination of ultimate tensile strength and electric conductivity of alloys via machine learning screening, Acta Materialia 200 803–810.

[34] Roy A., Babuska T., Krick B., Balasubramanian G.,(2020) Machine learned feature identification for predicting phase and Young’s modulus of low-, medium-and high-entropy alloys, Scripta Materialia 185 152–158.

[35] Kalinowski M., Lopes H., Teixeira A.F., da Silva Cardoso G., Kuramoto A., Itagyba B., et al., Lean R&D: An Agile Research and Development Approach for Digital Transformation, in: Morisio M., Torchiano M., Jedlitschka A. (Eds.) Product-Focused Software Process Improvement, Springer International Publishing, Cham, 2020, pp. 106–124.

[36] Guinan P.J., Parise S., Langowitz N.,(2019) Creating an innovative digital project team: Levers to enable digital transformation, Business Horizons 62(6) 717–727.

[37] de Lucas Ancillo A., Gavrila Gavrila S.,(2023) The Impact of Research and Development on Entrepreneurship, Innovation, Digitization and Digital transformation, Journal of Business Research 157 113566.

[38] Luo S.,(2022) Digital Finance Development and the Digital Transformation of Enterprises: Based on the Perspective of Financing Constraint and Innovation Drive, Journal of Mathematics 2022 1607020.

[39] Xu G., Li G., Sun P., Peng D.,(2023) Inefficient investment and digital transformation: What is the role of financing constraints?, Finance Research Letters 51 103429.

[40] Han H., Gu X.,(2021) Linkage Between Inclusive Digital Finance and High-Tech Enterprise Innovation Performance: Role of Debt and Equity Financing, 12. 10.3389/fpsyg.2021.814408 PMC8751544 35027906

[41] Zhang L., Fakieh B., Shang L.I.,(2021) Financial Management Of Asset-Liability Ratio Of Small-And Medium-Sized Enterprises In Dynamic Nonlinear System, Fractals 30(02) 2240064.

[42] Yuan R., Li C., Cao X., Li N., Khaliq N.,(2022) Research on the Influence of Mixed-Ownership Reform on Exploratory Innovation of SOEs: The Mediation Effect of Agency Conflict and Financing Constraint, SAGE Open 12(2) 21582440221093358.

[43] Jensen M.C., Warner J.B.,(1988) The distribution of power among corporate managers, shareholders, and directors, Journal of Financial Economics 20 3–24.

[44] Bai C.-E., Liu Q., Lu J., Song F.M., Zhang J.,(2004) Corporate governance and market valuation in China, Journal of Comparative Economics 32(4) 599–616.

[45] Jiang F., Cai W., Wang X., Zhu B.,(2018) Multiple large shareholders and corporate investment: Evidence from China, Journal of Corporate Finance 50 66–83.

[46] Rajagukguk J., Siagian H.L.,(2021) The Effect of Liquidity and Total Asset Turnover on Profitability: Research Study n Pharmaceutical Companies in Indonesia Stock Exchange, Ekonomis: Journal of Economics Business.

[47] Fairfield P.M., Yohn T.L.,(2001) Using Asset Turnover and Profit Margin to Forecast Changes in Profitability, Review of Accounting Studies 6(4) 371–385.

[48] Wang X., Li H., Pan T., Su H., Meng H., Material Quality Filter Model: Machine Learning Integrated with Expert Experience for Process Optimization, Metals, 2023.

