# Supplementary material for: Prediction of digital transformation of manufacturing industry based on interpretable machine learning
Source: PLoS One. 2024 Mar 29;19(3):e0299147. doi: 10.1371/journal.pone.0299147 (PMC10980183; doi:10.1371/journal.pone.0299147)
Supplement: S1 File — (DOCX) [file pone.0299147.s002.docx]

**Algorithm principle**

(1)Bagging algorithm

The Bagging (Bootstrap Aggregating) algorithm is an ensemble learning method that employs random sampling with replacement to generate multiple datasets. Each dataset is utilized to train a model using the same learning algorithm, and subsequently, the models are amalgamated to facilitate decision-making. Figure 1 exemplifies the principle of the Bagging algorithm, which encompasses the extraction of multiple training subsets from the original training dataset employing random sampling with replacement. The identical learning algorithm is employed on each subset to construct classification models, and subsequently, these models are consolidated to enable classification or regression decisions. For instance, classification tasks can implement voting, while regression tasks can adopt averaging. Consequently, when presented with new data samples, the ultimate prediction is obtained by inferring predictions from each model and calculating the average (or majority voting).

Extremely Randomized Trees, also known as Extra Trees, represent a variant of the Random Forest algorithm, in which the prediction for classification tasks relies on a voting mechanism. To classify a given sample, each decision tree provides a prediction, and the final class prediction is determined through voting. In contrast to conventional Random Forests, Extra Trees introduce additional randomization during the construction of decision tree nodes, specifically in the selection of features and split points. Rather than exclusively choosing the best split point from the candidate features, Extra Trees randomize the selection of candidate features and split points for each node. This randomization process promotes model diversity, diminishes feature dependency, and helps reduce model variance.


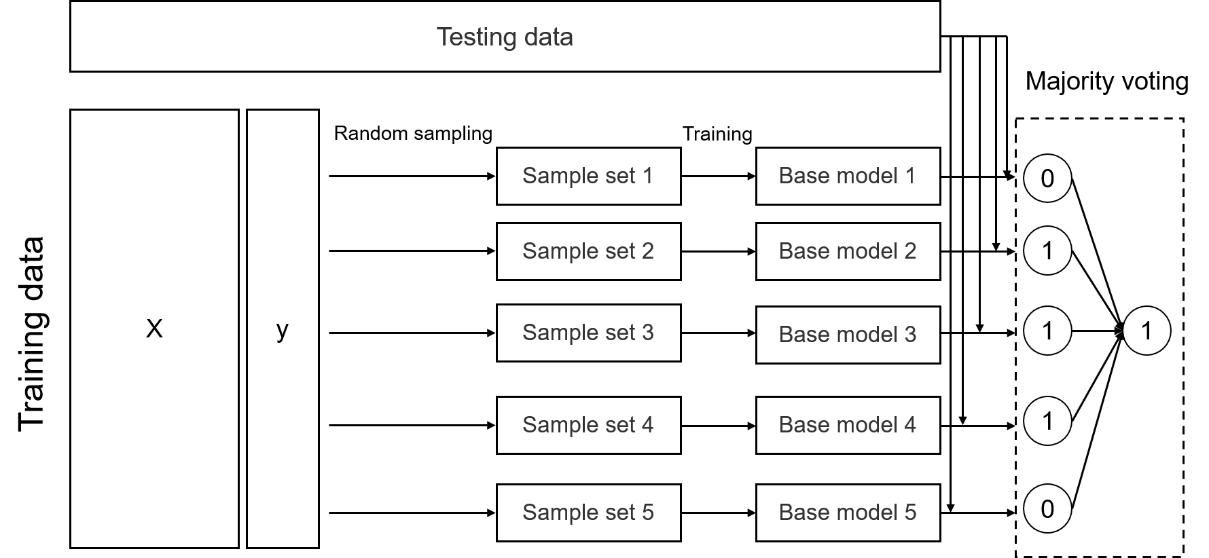


**Figure 1 Bagging algorithm Principle**

(2)Boosting algorithm

The Boosting algorithm is a powerful technique that can strengthen weak learners by iteratively training multiple models and gradually improving the overall predictive accuracy of the model. Unlike Bagging algorithms, which utilize independent models constructed using random sampling with replacement, Boosting algorithms achieve superior performance by repeatedly adjusting the weights of training samples and training weak learners to focus on misclassified samples. As depicted in Figure 2, the primary concept of the Boosting algorithm is to reweight the training samples based on the performance of previously trained weak learners. Misclassified samples are assigned greater weights to enable subsequent weak learners to better focus on these challenging instances. Through multiple rounds of weighted sampling and iterative training, the Boosting algorithm aims to minimize the overall prediction error and eventually produce a robust, accurate model.

The Gradient Boosting Machine (GBM) optimizes the loss function by employing gradient descent and adjusting the sample weights in each iteration. The residuals of the weak learner from the previous round serve as the target for training the subsequent weak learner. In the end, GBM combines multiple weak learners in a linear fashion, assigning specific weights to each, to construct the final robust learner.


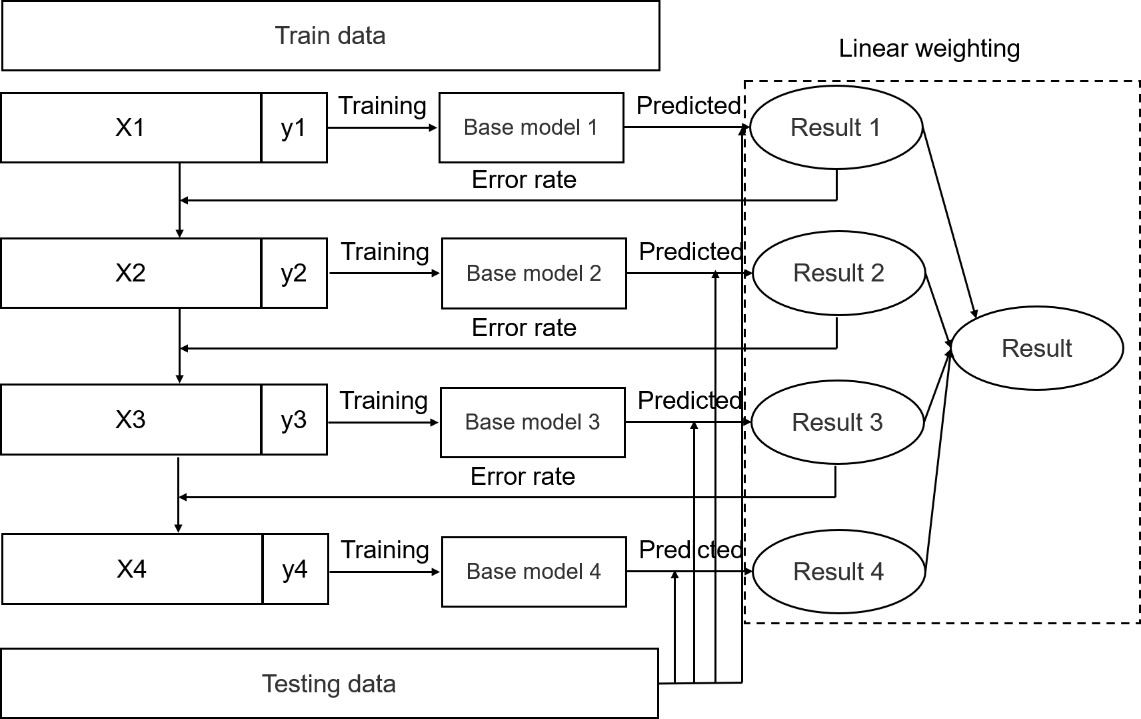


**Figure 2 Boosting algorithm principle**

(3)Support vector machine

Support Vector Machine (SVM) is a binary classification model that seeks to discover a hyperplane for effectively separating the training dataset while maximizing the separation between novel sample points on both sides of the hyperplane. The core principle behind SVM involves identifying an optimal hyperplane that maximizes the distance from samples of distinct classes. These specific samples, referred to as support vectors, play a crucial role. SVM accomplishes the classification task by constructing a decision boundary with the greatest margin to the nearest samples.

(4)Neural network

A neural network (Neural Network) is a computational model consisting of multiple neurons (or nodes). It is inspired by the interconnectedness and communication between neurons in the brain and can be used to simulate and solve various complex problems. As demonstrated in Figure 3, a neural network is composed of a vast number of interconnected artificial neurons. Each neuron receives input and calculates the final output by processing the input through weighted and activation functions. These neurons are hierarchically organized, with information flowing from the input layer to the output layer. Neural networks can automatically learn the relationship between input and output by adjusting the weights between neurons.


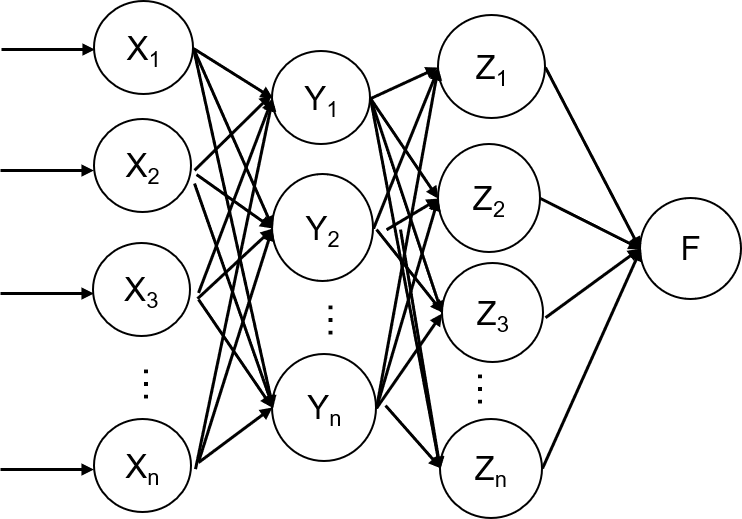


**Figure 3 Neural network structure**

**The meaning of C1 C2 C3 C4**

The C1 C2 C3 C4 codes are used to distinguish the four-level classification codes of the manufacturing industry in China. This classification is established by the National Bureau of Statistics of China and the Development Research Center of the State Council. Here are the specific meanings: C1 represents the major category, which is the highest level of the four-level code, indicating the primary classification of industrial sectors. It includes 33 categories, such as agricultural and sideline food processing, wood processing and furniture manufacturing, non-ferrous metal smelting and rolling processing, etc. C2 represents the medium category, which is the second level of the four-level code, indicating the detailed classification under the major industrial categories. There are 191 medium categories, such as pulp and papermaking, household appliance manufacturing, petroleum processing, coking, and nuclear fuel processing, etc. C3 represents the small category, which is the third level of the four-level code, indicating the detailed classification under the major medium categories. There are 1563 small categories, such as concentrated fruit juice and spice manufacturing, strawberry resin manufacturing, metal polishing/coating, machinery repair parts electroplating, etc. C4 represents the detailed category, which is the finest level of the four-level code, indicating the specific detailed classification under the major small categories. There are tens of thousands of detailed categories. For example, the manufacturing of reinforcing bars for reinforced concrete is a detailed category. The above four-level classification codes provide a specific division of the manufacturing industry, which helps the government and enterprises understand the development status and trends of different industries in industrial production, and provides references and guidance for decision-making.

**The specific explanation of the SHAP value**

The Shapley value, originally originated in game theory by Lloyd Shapley in 1953, is a method used to allocate payoffs in a game and determine the contribution of each player. The basic idea of the Shapley value is to measure the magnitude of each player’s contribution, which should depend on the different collaborations the player has with other players, i.e., the changes in payoffs caused by their presence in different coalitions. This definition of a contributor’s value can be represented by various mathematical formulas. Later, the Shapley value was introduced into social choice theory to address the problem of fair allocation of social welfare. Recently, the Shapley value has been introduced into the field of machine learning to explain the contribution of model predictions, i.e., the importance of features. SHAP (Shapley Additive Explanations) is a method developed based on the idea of the Shapley value to explain machine learning models. By applying the Shapley value to the features of the model, it can determine the contribution of each feature to the model predictions.

The working principle of SHAP (Shapley Additive Explanations) can be summarized as follows:

1. Calculate the model’s baseline value: Input all features into the model and calculate the average of the predicted values, which is the baseline value.

2. Sample the values of each feature: Sample the values of each feature and generate a dataset containing the different values of the feature to explore the model’s response to different values.

3. Calculate the model’s output value: Input the sampled dataset into the model and calculate the predicted value.

4. Calculate the impact of each feature on the predicted result: Compare the sampled dataset with the baseline value and calculate the impact of each feature on the predicted result using SHAP values.

5. Explain the prediction result: Based on the calculated SHAP values, explain the contribution of each feature to the predicted result and use visualization tools to display the explanation results.

SHAP (Shapley Additive Explanations) has the following applicability and limitations:

Applicability:

1. Generalizable: SHAP can be applied to a wide range of machine learning models, including linear models, tree-based models, and deep learning models.

2. Model-Agnostic: SHAP does not rely on specific model internals, making it applicable to any black box model.

3. Individual Explanations: SHAP provides individual feature contributions for each prediction, allowing for personalized explanations.

4. Consistent: SHAP guarantees that the sum of feature attributions equals the difference between the prediction and the average prediction.

Limitations:

1. Computational Complexity: SHAP can be computationally expensive, especially for high-dimensional feature spaces or large datasets.

2. Interpretability: While SHAP provides feature attributions, interpreting the results and understanding their implications may still be challenging.

3. Sampling Bias: The accuracy of SHAP values depends on the quality of the sampling technique used to approximate the feature space.

4. Feature Interactions: SHAP assumes feature independence, which may not hold in complex models with significant feature interactions.
